# Hybrid taguchi-grey relational analysis approach for optimizing cutter operational parameters in selective cauliflower harvesting

**DOI:** 10.1038/s41598-024-81323-z

**Published:** 2024-11-29

**Authors:** Ajay Kushwah, P. K. Sharma, H. L. Kushwaha, Brij Bihari Sharma, A. K. Shrivastava, Ramineni Harsha Nag, Manojit Chowdhury, Gopal Carpenter, Rashmi Yadav

**Affiliations:** 1 Research Scholar, Division of Agricultural Engineering, ICAR-IARI, New Delhi, 110012 India; 2 Principal Scientist, Division of Agricultural Engineering, ICAR-IARI, New Delhi, 110012 India; 3 Head, Division of Agricultural Engineering and Renewable Energy, ICAR-CAZRI, Jodhpur, 342003 India; 4 Department of Vegetable Science, College of Horticulture & Forestry, RLBCAU, Jhansi, 284003 India; 5 Dean, College of Agricultural Engineering, JNKVV, Jabalpur, 482004 India; 6 Scientist, ICAR-Central Institute of Agricultural Engineering, Bhopal, 462038 India; 7 Research Scholar, Water Technology Center, ICAR-IARI, New Delhi, 110012 India

**Keywords:** Selective cauliflower harvesting, Taguchi-based grey relational analysis, Chainsaw cutting mechanism, Cutting force, Mechanical engineering, Techniques and instrumentation, Electrical and electronic engineering

## Abstract

Cauliflower is an important winter crop grown in India, its curds are rich in nutritional profile, containing valuable minerals and vitamins. However, cauliflower harvesting is mainly accomplished by hands, which is time-consuming and requires a high labour force. On the other hand, most developed cauliflower harvesters are once over or single pass type, which harvests all plants irrespective of their maturity. So, the selective harvester could improve the cauliflower curds yield, and then decrease the labour requirement. To improve the cutting performance of the selective cauliflower harvester, the working parameters of the chainsaw cutting mechanism need to be considered and optimized. This research investigates the impact of cutting height, feed (push) force, and cutting speed on the efficiency of the cutter during harvest. The Taguchi approach, together with grey relational analysis (GRA), was employed to identify the most favorable combination of operational parameters. In addition, the variance analysis was conducted to statistically examine the impact of multiple parameters. The findings indicated that the feed force was the major parameter that influenced the cutting force, splitting failure levels, and cutting time. The most effective parameter combination consisted of a cutting height of 15 mm, a feed force of 10 N, and a cutting speed of 5 m/s. The grey relational grade of the ideal parameter combination has shown a 0.322 increase in comparison to the grade achieved with the initially selected parameter combination. This setting was further incorporated in the developed selective cauliflower harvester to improve the performance of its cutting mechanism.

## Introduction

Horticulture has a pivotal role in enhancing global health and fostering economic progress in nations. India is the second largest global producer of fruits and vegetables in the field of horticultural production^1^. Out of the total cultivated land of 194 million hectares in India, around 8% is specifically used for growing fruits and vegetables. This sector employs around 58% of the Indian population, either directly or indirectly^2^. The horticulture production in India for 2022-23amounted to a significant volume of 355.5 million tonnes, covering an area of 28.4 million hectares^3^. India, as the second largest vegetable producer, recorded a vegetable production of 212.54 million tonnes across an area of 11.30 million hectares^4^. Specifically, in cauliflower (Brassica oleracea L. var. botrytis) production, India holds the second position globally, trailing only behind China. The cauliflower cultivation area in India spans 0.48 million hectares, resulting in a substantial production volume of 9.53 million metric tonnes during the 2021-22period^5^.

In India, the traditional method of harvesting cauliflower primarily depends on manual labour and hand tools, such as sickles. However, this approach is filled with constraints, particularly worsened by a lack of available workers during peak times, leading to delays in operations and subsequent decreases in productivity^6^. Several researchers have attempted to create specialized harvesters for cole crops that can harvest all curds regardless of their maturity stage^7–12^. The size of cauliflower curd acts as a good predictor of its maturity^13,14^. However, harvesting cauliflower poses a particular problem, as the white heads (curds) in the field do not grow concurrently, with just 50% attaining maturity after 60 to 70 days of planting^15,16^. This needs pickers to patiently analyze each cauliflower to establish the best ripened harvesting stage, often needing four to five trips across the field. This labor-intensive and physically demanding task might provide substantial obstacles for the participating laborers^17^. Nonetheless, limited emphasis has been paid towards selective harvesting approaches in recent literature^18–20^. The cutting mechanism is an essential component of the harvesting process for cole crops such as cauliflower, broccoli, and cabbage. Several cutting techniques have been developed to enhance the precision and efficiency of harvesting cole crops. Hamdy (1962)^21^conducted groundbreaking research on developing a cabbage harvester. As part of this work, specialized lifter shoes were created, equipped with two types of disc blades - plain and serrated. These knives were designed to efficiently cut the roots of the plants. The cole crop harvesting process involved using a hydraulic motor-driven circular crosscut saw blade and a counter-rotating disc cutter^10–12,22,23^. Furthermore, a hydraulic motor-driven long knife with an impact-type mechanism was employed for the purpose of selectively harvesting cauliflower^24^. In their study, Klein et al. (2019)^25^introduced a novel slicing-cutting mechanism that utilized an abrasive wire in a cutting end effector to effectively severe the cauliflower stem. An innovative chain saws cutting mechanism was created specifically to selectively harvest cauliflower. Prior research has demonstrated that the cutting height has an impact on the amount of force used to cut the stalk. The cutting force was found to be the lowest when the cutting position was within a range of 40 mm from the bottom of the curd^26,27^. The cutting position has an impact on both the occurrence of splitting failure, which is a type of damage that happens along the direction of the fibres, and the duration of the cutting process^25,28^. The chain saw cutting performance i.e. cutting force and cutting time was impacted by the push (feed force) and cutting speed when operating on the timber^29,30^. These operational characteristics of each mechanism need to be tuned for improved cutting performance of the developed harvesters. Diverse optimization techniques, such as Response Surface Methodology (RSM), Multi-Objective RSM, Particle Swarm Optimization (PSO), Gene Expression Programming (GEP) in conjunction with PSO, and Grey Relational Analysis integrated with Principal Component Analysis (PCA), have been utilized to ascertain optimal machining parameters to reduce lubrication needs^31–35^. Optimization approaches transcend manufacturing; for instance, refining the shape and weight of helmets can markedly diminish damage risks^36^. Although there are several studies on cutter working parameter optimization of other crops with different optimization approaches i.e. factorial completely randomized design, response surface methodology, Taguchi and Taguchi-based grey relational analysis, but few on cauliflower harvesters^12,28,37,38^. The combination of the Taguchi methodology with GRA is currently considered a valuable technique for multi-objective optimization. This strategy has the potential to decrease the quantity of tests and computation time needed^39^.

The conventional Taguchi approach can be utilized to optimize one particular response parameter^40^. However, the Taguchi technique does not consider the optimization of many responses. Currently, the integration of the Taguchi technique, grey relational analysis (GRA), and analysis of variance (ANOVA) has emerged as a very effective and potent tool for addressing engineering issues^41–44^. Du et al. (2023)^37^improved the working parameters such as tooth top width, tooth root width, tooth depth, teeth space, and cutting speed ratio of reciprocating type cutter bar for tea leaf harvesting using Taguchi-based grey relational analysis. Gopalsamy et al. (2009)^45^used the Taguchi approach, GRA, and ANOVA to study the performance characteristics of machining process parameters such as cutting speed, cut width, and depth to determine the ideal process parameters. This analytical method has been extensively utilized to assess the thermal transfer efficacy of direct oil cooling systems in electric vehicles^46^and to optimize the performance of two-stage gearboxes^47^. However, there is no research on the optimization of the approaches stated above for the working parameters of the innovative chainsaw-cutting mechanism utilized in selective cauliflower harvesting in the literature.

This study utilized the Taguchi with GRA technique to optimize the chainsaw-cutting mechanism’s operating parameters on a selective cauliflower harvester. The cutting height, feed force, and cutting speed were considered working (independent) parameters whereas the cutting force, splitting failure levels, and cutting time were the optimizing (response) indexes. The Taguchi-based GRA technique was applied to obtain the relevance of influence parameters and the ideal set of operation parameters. After that, this precise combination of operational characteristics of the cutting mechanism was utilized further to develop a selective cauliflower harvester.

## Materials and methods

### Description of experimental setup and measurements

#### Chain saw cutting mechanism

**Fig. 1:**
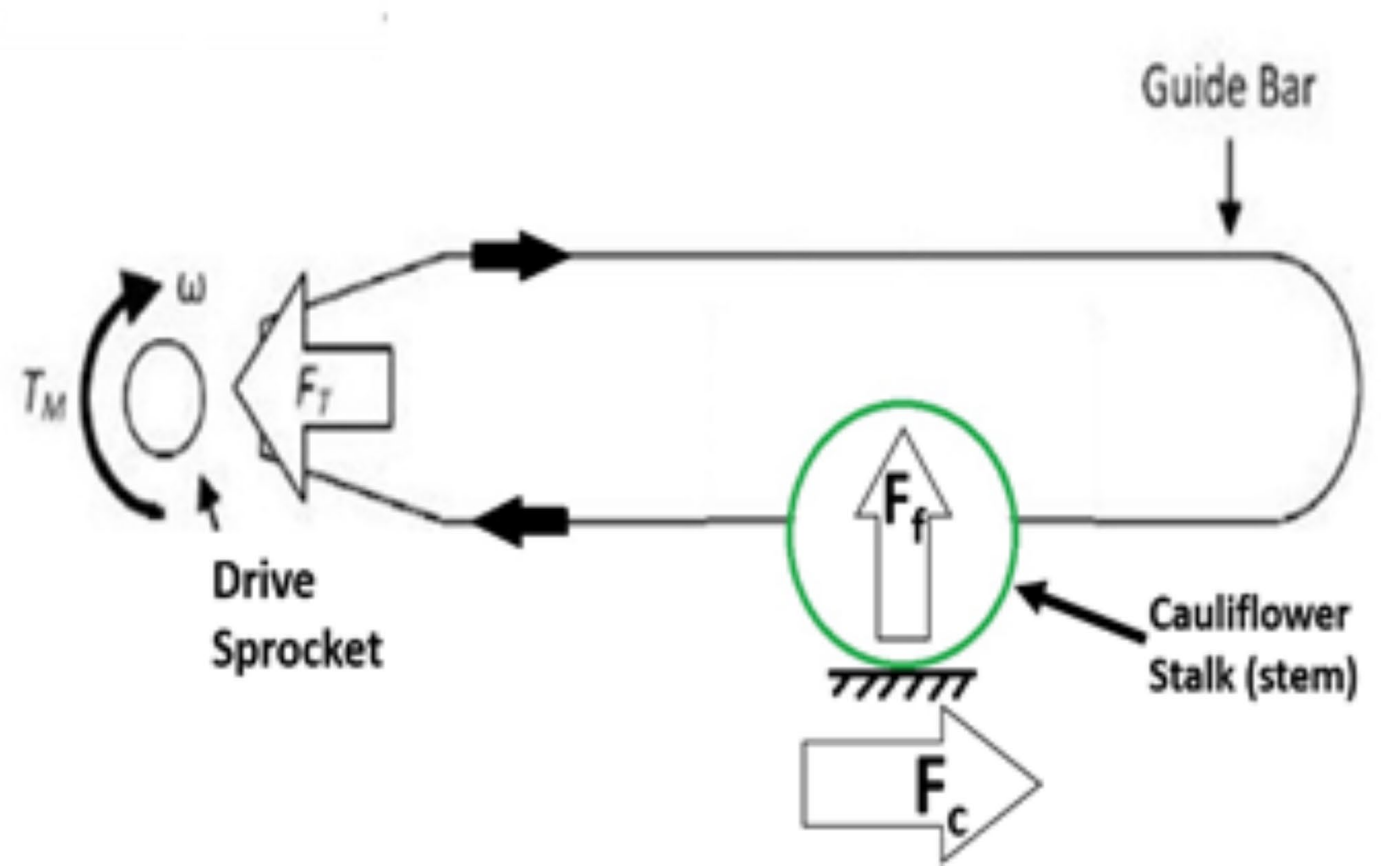
A schematic of a chainsaw cutting a stalk (cross section) showing the measured cutting parameters of chain velocity, feed force, and cutting force.

The chain saw cutting mechanism was used in the selective harvesting system. The cutting mechanism was composed of a cutting chain, guide bar, and drive sprocket. The cutting chain has a length of 300 mm with a chain pitch of 9.525 mm and 45 drive links. The driving links connected with the driving sprocket (6 teeth) drive the chain and also slide into a notch in the guide bar, assuring the motion of the saw chain along the edge of the guide bar. The principles underlying the cutting parameters of a chainsaw are shown in Fig. 1. During the cutting process, the cutting force (F_c_) was exerted on the cauliflower stalk parallel to the guide bar, while the feed force or push force (F_F_) was applied perpendicular to the guide bar. The supplied torque (T_M_) was transmitted to the drive gear to power the saw chain, and ω represented the angular speed of the drive gear (sprocket). The cutting speed (V_c_) represented the speed at which the saw chain moved during cutting. When the chain was not in touch with the workpiece, the non-cutting chain tension was referred to as the force (F_T_). The chain tension of 89 N (± 5 N) was required to prevent the deposition of the cutting chain from the guide bar^29^.

#### Developed experimental setup

**Fig. 2:**
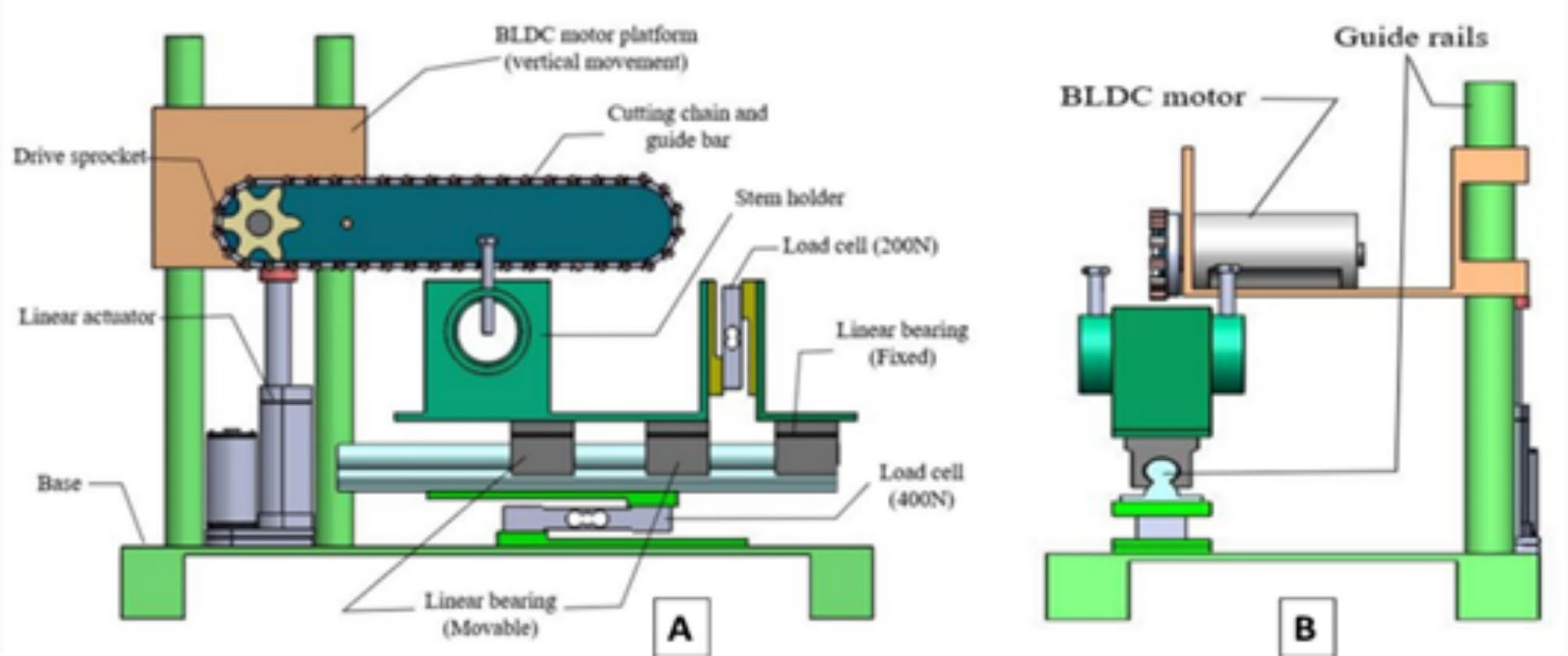
Experimental setup for cutting force measurement (**A**) Front view (**B**) Side view.

**Fig. 3:**
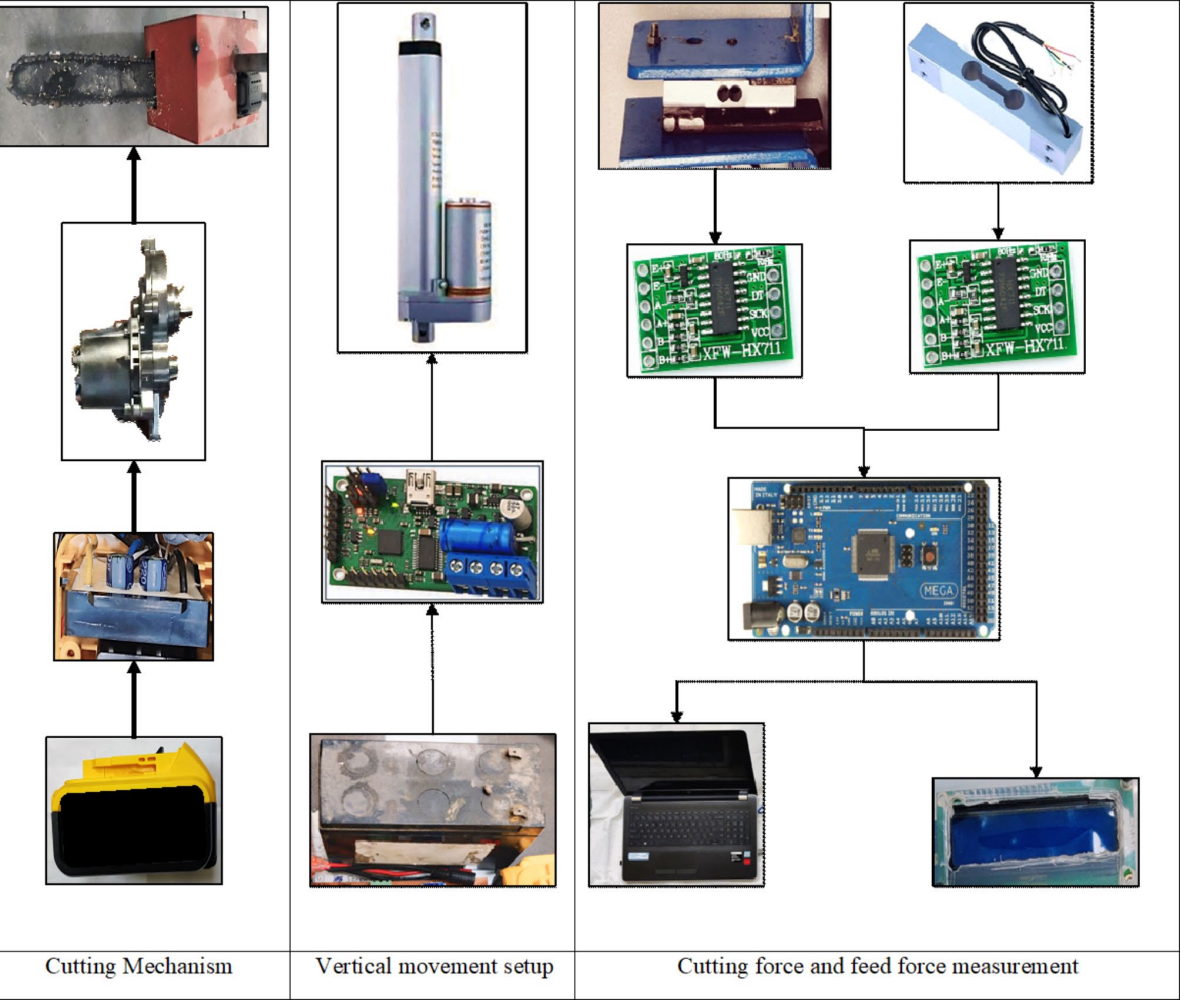
Working of different subsystems.

**Fig. 4:**
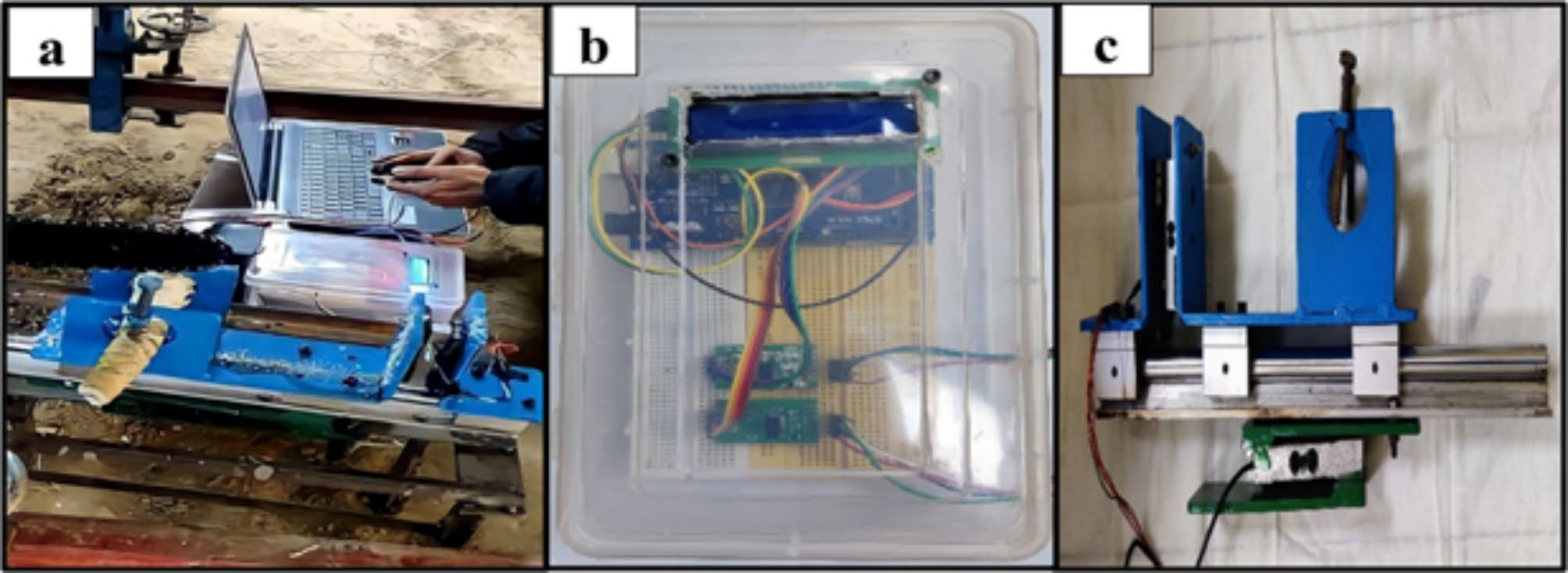
(**a**) Cutting force measurement (**b**) Data acquisition system (**c**) Stem holder with load cells.

An experimental setup was developed to enable the dynamic measurement of cutting forces (Fig. 2). The setup comprised four major parts: a power head, a motion system, a work (stalk) holding system, and a data acquisition system (Figs. 3 and 4). The power head propelled the saw chain, by utilizing a drive sprocket and a geared 350 W BLDC (brushless direct current) motor with a maximum speed of 7,000 RPM and was effectively controlled through a pulse width modulation (PWM) brushless motor controller driver. It was powered by an 18 V Li-ion battery (Fig. 3). The chainsaw cutting speed was measured with the help of the rotational speed of the drive sprocket. The rotational speed of the sprocket was accurately measured using an optical tachometer. The motor was securely fastened on the platform next to the guiding bar. The platform itself exhibited the capability to execute vertical movements at a maximum speed of 20 mm/s, facilitated by a high-performance 100 N linear actuator, powered by a 12 V lead acid battery. The actuator’s linear speed was controlled using a basic motor controller, enabling accurate control of the feed force exerted on the stalk (Fig. 3). To measure this exerted feed force, a highly sensitive 400 N load cell was employed (Fig. 4c). However, the stalk was firmly held within a specially designed stalk holder. This holder was granted the ability to move horizontally by incorporating two linear bearings at its base. Nevertheless, the extent of motion was significantly limited by a stationary linear bearing, which was in turn linked to a load cell with a force capacity of 200 N. As the working chain saw came into contact with the stalk, the chain tried to pull the stalk along with the holder (Fig. 4a). But, the movement of the holder was restrained by the fixed linear bearing, resulting in the recording of the tension force via the 200 N load cell (Fig. 4c). This recorded tension force represented the essential cutting force required to sever the stem. The data acquisition system effectively captured and showed the output from the load cells through the utilization of a microcontroller (Arduino Mega 2560), load cell amplifier modules (HX711), 16 × 2 LCD, and a personal computer (Figs. 3 and 4b).

**Fig. 5:**
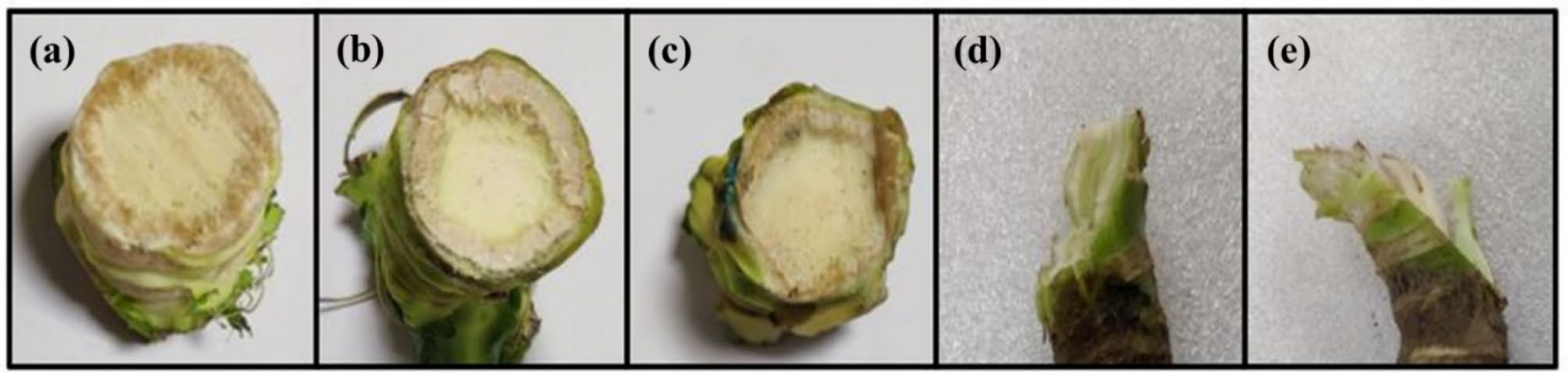
Splitting failure levels (**a**) None splitting (**b**) Slight splitting (**c**) Medium splitting (**d**) High splitting (**e**) Severe splitting.

The working parameters (independent parameters) such as cutting height (CH), feed force (FF), and cutting speed (CS) were considered for investigating the effect on responses such as cutting force (CF), splitting failure levels (SFL), and cutting time (CT). The cutting height is defined as the distance from the top leaves (bottom of the cauliflower curd) measured along the cauliflower stalk. The feed force (push force) and cutting speed were controlled by a linear actuator and a BLDC motor controller driver, respectively. The cutting force was measured using an embedded system and displayed on an LCD screen, while also being recorded on a personal computer (Fig. 3). The splitting failure refers to the damage of the stem along the direction of the fiber^28^. The splitting failure levels (SFL) were categorized based on the observed splitting thickness into five different levels: extreme splitting (level 5: more than 5 mm); high splitting (level 4: 5 –3 mm), medium splitting (level 3: 3 –1 mm), slight splitting (level 2: 1 –0 mm), and no splitting (level 1) as shown in Fig. 5. The cutting time is defined as the time required to complete the cutting process.

### Hybrid taguchi-grey relational analysis

To address the collinearity issue among the operating parameters, the Taguchi technique in conjunction with grey relational analysis (GRA) was utilized to discover the ideal combination of these parameters^37,43^.

#### Taguchi method

The Taguchi technique was developed to create orthogonal arrays to assess the impacts of numerous parameters. This experiment examined three working parameters with three levels each based on the Taguchi method. The independent parameters and their relative levels are provided in Table 1. An initial test was conducted using the parameter combination A2B2C2, which consisted of a cutting height of 30 mm, a feed force of 20 N, and a cutting speed of 3.5 m/s. Taguchi L_9_ orthogonal array was elected for this investigation (Table 2), which generated experimental settings based on the chosen orthogonal array^39,43,48^. The Minitab software was used for this analysis (Minitab version 20.3 © 2023 Minitab, LLC).

**Table 1:** Plan of experiment for selection of optimum setting of input parameters at laboratory.

Independent variables	Code	Levels of variables			Response variables
		1	2	3	
Cutting height (mm)	A	15	30*	45	Cutting force (CF, N)
Feed force (N)	B	10	20*	30	Splitting failure levels (SFL, level)
Cutting speed (m/s)	C	2	3.5*	5	Cutting time (CT, s)

*Initial operating setting- A2B2C2.

**Table 2:** Experimental runs and their responses with the s/n ratio.

Trial no.	Independent Variables			Response Variables			S/*N* ratios (dB)		
	CH, mm	FF, *N*	CS, m/s	CF, *N*	SFL	CT, s	CF	SFL	CT
1	1	1	1	54.14	1	1.7	−34.67	0.00	−4.61
2	1	2	3	49.98	2	1.51	−33.98	−6.02	−3.58
3	1	3	2	57.51	5	1.77	−35.19	−13.98	−4.96
4	2	1	2	54.4	1	1.72	−34.71	0.00	−4.71
5	2	2	3	54.01	2	1.55	−34.65	−6.02	−3.81
6	2	3	1	64.7	5	1.86	−36.22	−13.98	−5.39
7	3	1	3	53.31	1	1.72	−34.54	0.00	−4.71
8	3	2	1	64.5	4	1.75	−36.19	−12.04	−4.86
9	3	3	2	63.24	5	1.86	−36.02	−13.98	−5.39

#### Analysis of the signal-to-noise ratio

The Taguchi methodology utilizes the signal-to-noise ratio (S/N) as a key statistical metric. It is calculated using a logarithmic function to evaluate the ratio between the signal (mean) value and the noise (standard deviation) that exists among the variables^38^. The noise component is predominantly attributed to uncontrollable factors associated with the experimental setup, necessitating their mitigation to diminish the noise levels and subsequently minimize the variance observed in the experimental or actual variables. Higher S/N ratios denote reduced noise levels and optimal working parameters, consequently yielding superior responses. The evaluation of response characteristics is based on three separate categories of signal-to-noise ratios (S/N ratios): larger-the-better (LB), nominal-the-better (NB), and smaller-the-better (SB). These ratios are calculated as follows^37^:

1 $$\:\mathbf{L}\mathbf{B}:\:\:\:\frac{S}{N}=-10\times\:{log}_{10}\left(\frac{1}{n}\sum\:_{i=1}^{n}\frac{1}{{Y}_{i}^{2}}\right)$$

2 $$\:\mathbf{N}\mathbf{B}:\:\:\frac{S}{N}=-10\times\:{log}_{10}\left(\frac{1}{n}\sum\:_{i=1}^{n}{\left({Y}_{i}-{Y}_{0}\right)}^{2}\right)$$

3 $$\:\mathbf{S}\mathbf{B}:\:\:\frac{S}{N}=-10\times\:{log}_{10}\left(\frac{1}{n}\sum\:_{i=1}^{n}{Y}_{i}^{2}\right)$$

Where ‘n’ represents the total number of experiments conducted, ‘i’ denotes the specific experiment number, ‘Y_i_’ signifies the response value at the ‘i^th^’ experiment, and ‘Y_0_’ denotes response value at baseline (0th ) experiment. The choice of larger-the-better (LB), nominal-the-better (NB), and smaller-the-better (SB) criteria depends on whether the goal is to maximize, average, or minimize the response, respectively. In the current investigation, the response characteristics under consideration encompass cutting force, splitting failure levels, and cutting time, which necessitates optimization with a focus on smaller-the-better quality attributes.

#### Grey relational analysis method

Single-response optimization problems are especially well-suited for the Taguchi orthogonal array approach. However, Grey Relational Analysis (GRA) is highly effective in solving complex optimization problems that involve many objectives by deriving the Grey Relation Grade (GRG). The GRG is crucial to determine the best arrangement of several working parameters to attain specific quality attributes. Within the GRA framework, initial data preprocessing techniques are crucial to address issues of scale, unit, and target. Previous works showed the use of signal-to-noise (S/N) ratio normalization within GRA to produce the dimensionless sequence of data^42^. Subsequently, the original response-to-S/N ratio is normalized within the 0–1 range to facilitate subsequent analysis. Moreover, distinct normalization approaches have been described for each response variable in Eqs. (4)-(5)^41^.

4 $$\:\mathbf{L}\mathbf{B}:\:\:\:\:\:{x}_{i}\left(k\right)=\frac{{y}_{i}\left(k\right)-miny\left(k\right)}{maxy\left(k\right)-miny\left(k\right)}$$

5 $$\:\mathbf{S}\mathbf{B}:\:\:\:\:\:{x}_{i}\left(k\right)=\frac{maxy\left(k\right)-{y}_{i}\left(k\right)}{maxy\left(k\right)-miny\left(k\right)}$$

Where x_i_(k) = normalized value for k^th^ response at the i^th^ experiment;

y_i_(k) = original value for k^th^ response at the i^th^ experiment;

min y(k) = smallest value of y_i_(k) for the k^th^ response in all experiment;

max y(k) = largest value of y_i_(k) for the k^th^ response in all experiment.

The values of the loss function were obtained by Eq. (6)

6 $$\:{{\Delta\:}}_{i}\left(k\right)=1-{x}_{i}\left(k\right)$$

Next, the computation of the grey relational coefficient (GRC) was carried out utilizing the loss function values, as described by the following Eq. (7):

7 $$\:{GRC}_{i}\left(k\right)=\frac{{{\Delta\:}}_{min}\left(k\right)+\lambda\:{{\Delta\:}}_{max}\left(k\right)}{{{\Delta\:}}_{i}\left(k\right)+\lambda\:{{\Delta\:}}_{max}\left(k\right)}$$

Where GRC_i_(k) = value of grey relational coefficient for k^th^ response at the i^th^ experiment;

Δ_i_(k) = difference of absolute value between the targeted sequence (i.e. the maximum value of x_i_(k) for k^th^ response) and comparison sequence (i.e. value of x_i_(k) for k^th^ response at the i^th^ experiment);

Δ_min_(k) = smallest value of Δ_i_(k) for the k^th^ response in all experiment;

Δ_max_(k) = largest value of Δ_i_(k) for the k^th^ response in all experiment;

λ = distinguishing coefficient in the range of 0 to 1 (set as 0.5 in this study).

In the final step, GRG for each experiment was calculated by summing the all GRC corresponding to each experiment divided by total number of responses as described by the following Eq. (8):

8 $$\:{GRG}_{i}=\frac{1}{m}\sum\:_{m=1}^{m}{GRC}_{i}$$

Where GRG_i_ = grey relational grade for each experimental run;

m = total no. of response (3 in this study).

#### ANOVA of grey relational grade

ANOVA, a statistical approach, serves to detect the influence of working parameters on a response, providing a numerical assessment evaluation of their contribution. This strategy becomes vital as the Taguchi experimental methodology alone cannot determine the effect of specific parameters on the entire process. The F-test within ANOVA assesses the importance of factors controlling the test findings. For a 95% confidence interval, the estimated p-values should be less than 0.05%, then the variables and interactions are significant. Within ANOVA, the adjusted correlation coefficient (adj. R^2^) is utilized to verify the validity of the fitted model. Adjusted R^2^estimates the percentage of variation in response variables exclusively explained by independent factors significantly impacting them. Moreover, to verify the suitability of generated models in describing experimental outcomes, it is preferred that both R^2^and adj. R^2^exhibits high values and is closely matched^49^.

#### Confirmation test

Once the optimal combination of parameters and their corresponding levels were identified, a verification test was deemed necessary to examine the results^43,45^. The estimated Grey Relation Grade (GRG) value at the optimal level of working parameters can be calculated as follows:

9 $$\:{\gamma\:}_{e}={\gamma\:}_{a}+\sum\:_{i=1}^{n}({\gamma\:}_{i}-{\gamma\:}_{a})$$

Where, $\:{\gamma\:}_{e}$ is the estimated GRG; $\:{\gamma\:}_{a}$ is the mean of total GRGs; $\:{\gamma\:}_{i}$ is the mean GRG at the optimal level; n is the number of operating parameters (Here, *n* = 3).

## Results and discussion

### Analysis of s/n ratio

The experimental data was analyzed using GRA to calculate the S/N ratios for Taguchi L9. In this study, the “smaller-the-better” characteristic was chosen for the cutting force, SFL, and cutting time, indicating that lower values were desired for optimal performance. The S/N ratios were determined using Eq. (3) and are presented in Table 2. Then, the mean S/N ratio was determined for different levels (1, 2, and 3) of each parameter (A, B, and C). The best level of response could be expressed by the greatest mean S/N ratio^50^. The mean S/N ratio for all responses is presented in Tables 3, 4 and 5.

#### Cutting force

**Fig. 6:**
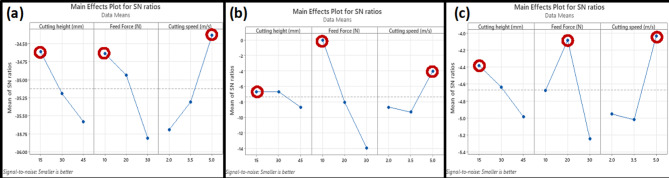
Main effects plot of S/N ratios for (**a**) cutting force (**b**) SFL (**c**) cutting time.

Table 3 presents the mean S/N ratio for the cutting force. The last row of the table (Rank) indicates the sequence of working parameters that have an impact on the cutting force, with cutting speed (C) having the greatest influence, followed by feed force (B), and then cutting height (A). The delta value represents the difference between the maximum and minimum mean S/N values. The best combination of operating parameters for minimizing the cutting force was found to be A1B1C3, corresponding to a cutting height of 15 mm, a feed force of 10 N, and a cutting speed of 5 m/s (Fig. 6a).

It was observed that an increase in cutting speed resulted in a decrease in cutting force, whereas cutting force exhibited an upward trend with increasing feed force and cutting height. Upon inspecting the cauliflower stalk, it was observed that the diameter of the stalk gradually increased from the root side to the curd side. For efficient cutting, the cutting region was selected to be 15 to 45 mm from the curd bottom^27^. Analyzing the cross-section of the cauliflower stalk, a fiber layer of approximately 3 to 5 mm thick was noticed, arranged in a circular pattern around the inner core, providing structural strength to the plant. The inner core exhibited a softer texture compared to the fiber layer. The fiber content showed an increasing trend from the curd side toward the root side. During the cutting process, when the cutting chain came into contact with the stalk in the cutting zone, a momentary peak in cutting force was observed, followed by a return to normal levels. This peak was a result of the initial contact of the cutting chain with the outer fiber layer, which offered higher resistance due to its strength and was also influenced by the transient effect^51^. As the cutting height increased (measured from the curd bottom) or the diameter decreased, the fiber layer thickness also increased, resulting in higher cutting forces encountered during the cutting process. Similar observations have been reported by other researchers in studies of different cole crops^26,27,52^. The devised cutting mechanism revealed a much lower cutting force required (46.7 N) compared to impact cutting (94.24 N) and blade slicing (398.8 N) for cauliflower^9,24^. Although abrasive wire slicing displayed an even lower cutting force (10 N), it took around 10 s to complete the cut, making it considerably slower than the designed mechanism^25^. An increase in the feed force was observed to correspond with a rise in cutting force, primarily due to the higher loading exerted on the cauliflower stalk during the cutting process. The increased loading on the stalk resulted in a slight reduction in cutting speed, which, in turn, led to higher cutting resistance and subsequently, an increase in cutting force. This finding aligns with the results reported by Maciak et al. (2018)^29^in their study of cutting wood using a chain saw cutting mechanism. When the cutting speed is increased, the cutting force decreases. This is because the higher cutting speed reduces the cutting resistance encountered by the cutting chain, resulting in a decrease in the force required for cutting. The same relationship between cutting speed and cutting force has been reported in studies^27,29,52^. Also, a higher cutting speed allows the cutting teeth of the chain to penetrate the cauliflower stalk more smoothly and efficiently, reducing the overall force required for the cutting process.

Also, the analysis of variance indicated that cutting height, feed force, and cutting speed significantly influenced cutting force (Table 6). Conversely, the interactions between the independent parameters did not exert a significant effect on cutting force. The coefficient of determination (R^2^) for the best-fit regression model was determined to be 0.998, with an adjusted R^2^ of 0.993. These values suggest that the model accounts for 99.8% of the variability in cutting force, indicating its high explanatory power and robustness.

**Table 3:** Response tables for S/N ratios of cutting force.

Smaller is better			
Level	A, Cutting height (mm)	B, Feed force (*N*)	C, Cutting speed (m/s)
1	**−34.61**	**−34.64**	−35.69
2	−35.19	−34.94	−35.31
3	−35.58	−35.81	**−34.39**
Delta	0.97	1.17	1.31
Rank	3	2	1

**Table 4:** Response table for S/N ratios of splitting failure.

Smaller is better			
Level	Cutting height (mm)	Feed force (*N*)	Cutting speed (m/s)
1	**−6.67**	**0.00**	−8.67
2	−6.67	−8.03	−9.32
3	−8.67	−13.98	**−4.01**
Delta	2.01	13.98	5.31
Rank	3	1	2

**Table 5:** Response table for S/N ratios of cutting time.

Smaller is better			
Level	Cutting height (mm)	Feed force (*N*)	Cutting speed (m/s)
1	**−4.38**	−4.68	−4.95
2	−4.64	**−4.08**	−5.02
3	−4.99	−5.25	**−4.03**
Delta	0.61	1.16	0.99
Rank	3	1	2

**Table 6:** Mean sum of square of responses obtained from different working parameters.

Source	df	Cutting force	Splitting failure levels	Cutting time
Cutting height (mm)	2	31.78^*^	0.44^NS^	0.010^*^
Feed Force (N)	2	29.24^*^	9.38^*^	0.014^*^
Cutting speed (m/s)	2	38.97^*^	0.71^NS^	0.009^*^
Error	2	0.21	0.18	0.000
Total	8			
Model Summary		S: 0.4553 R^2^: 99.83% R^2^(adj): 99.31%	S: 0.4216 R^2^: 98.68% R^2^(adj): 94.71%	S: 0.0194 R^2^: 99.35% R^2^(adj): 97.39%

* Significant; NS-Non-significant; df- Degree of freedom.

#### Splitting failure level

Table 4 displays the mean S/N ratio for the splitting failure level. The range analysis showed that each factor’s significance on SFL by order was B > C > A. The optimal combination of working parameters for minimizing the SFL was also A1B1C3 (Fig. 6b).

The crude fiber content declined as the cutting height decreased and reached its lowest near the curd resulting in the decrease of SFL. This finding aligns with the results reported by^26,28^. The SFL is increased with an increase in the feed force. The reason for this is that increasing the feed force leads to an increase in the vertical cutting force, which is the main factor responsible for SFL. Additionally, the enhanced vertical cutting force or feed force leads to a bigger contribution of shear force. Therefore, the increased shear stress ultimately increases the occurrence of splitting failure. SFL also decreased with the increase in cutting speed, although the difference was not statistically significant. The reason behind this trend was that higher cutting speeds enable the cutting teeth of the chain to penetrate the cauliflower stalk more smoothly and efficiently, leading to a more continuous cutting process. This continuous cutting process resulted in a reduction in SFL^28^.

Analysis of variance (ANOVA) revealed that only feed force had a significant influence on splitting failure (Table 6). In contrast, cutting height, cutting speed, and the interactions between the independent parameters were found to have no significant effect on SFL. The regression model exhibited a coefficient of determination (R^2^) of 0.986 and an adjusted R^2^ value of 0.947.

#### Cutting time

Table 5 presents the mean S/N ratio for the cutting time. The range analysis showed that B > C > A was the order in which each factor’s relevance on cutting time was greatest. It was discovered that A1B2C3 was the ideal set of operating settings for reducing the cutting time (Fig. 6c).

The cutting time was found to increase with cutting height, as higher cutting height is associated with more fiber content in the cauliflower stalk. The increased strength of the fiber content requires more time to cut through, leading to longer cutting time. These findings are in line with the results reported by^26,27^. Initially, the cutting time showed an increment in feed force led to a decrease in cutting time due to an increase in the cutting rate, resulting in a shorter cutting time. However, a continuous increase in feed force beyond a certain point led to an increase in cutting time. This was because the higher loading on the chain resulted in a reduction in chain speed, leading to a longer cutting time. Also, it was observed that an increase in cutting speed led to a decrease in cutting time^29^. Analysis of variance (ANOVA) revealed that cutting height, feed force, and cutting speed had a significant influence on cutting time Also, the R^2^and adjusted R^2^ values were close to each other (Table 6).

Based on the above analysis, it is evident that the optimal combinations of parameters differ for different responses. The optimal combinations were found to be A1B1C3 for the cutting force, A1B1C3 for the SFL, and A1B2C3 for the cutting time. The GRA method was used to optimize multiple objective responses to determine the optimal combination of parameters.

### Multi-response optimization using grey relational analysis

**Fig. 7:**
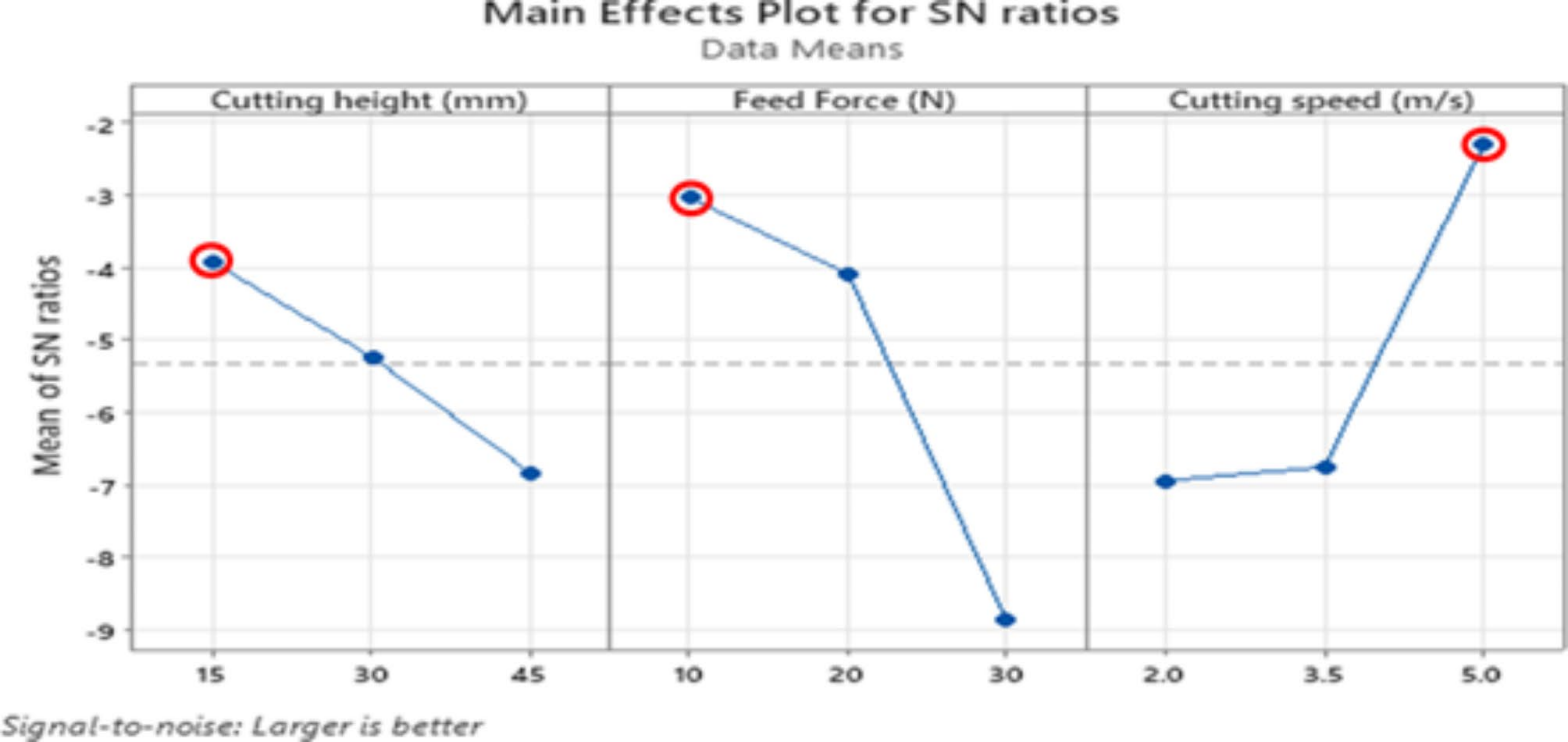
Main effects plot of S/N ratios for GRG.

Grey relational analysis (GRA) is commonly employed to analyze complex problems involving multiple response variables with various factors. The goal was to minimize the cutting force, splitting failure level (SFL), and cutting time to achieve optimal cutting mechanism performance. To accomplish this, the signal-to-noise (S/N) ratios were first normalized using Eq. 5 provided in Table 7. Then, the grey relational coefficient (GRC) and grey relational grade (GRG) values were calculated based on the normalized S/N ratios utilizing Eqs. 7–8. According to Pan et al. (2007)^53^, there is a positive correlation between the GRG value and the quality of the numerous output characteristics. In other words, a higher GRG value indicates better performance. These values were used to conduct GRA, which resulted in assigning ranks from 1 to 10 to interpret the GRG values relative to the target or optimal solution (Table 7). Through this analysis, experiment number 2 was identified as having the highest GRG value, suggesting its proximity to the desired optimal solution^43^. Furthermore, Table 8 displayed the means of the GRG values for each level of the parameters. The delta value (max-min) of the grey correlation served as an indicator of the factors’ influence. By ranking the factors in Table 8, their order of significance as follows: B > C > A. Ultimately, the best combination of operating parameters, which simultaneously minimized the cutting force, SFL, and cutting time, was found to be A1B1C3. This combination corresponded to a cutting height of 15 mm, a feed force of 10 N, and a cutting speed of 5 m/s. This configuration yielded the highest GRG value, signifying its optimal performance in the cutting mechanism (Fig. 7).

**Table 7:** Normalization and rank of GRG with S/N ratios for multiple responses.

Trial no.	Normalized value of S/*N* ratios			Grey relational co-efficient (GRC)			Grey relational grade (GRG)	Rank	S/*N* ratios for GRG
	CF	SFL	CT	CF	SFL	CT			
1	0.717	1.000	0.457	0.639	1.000	0.479	0.706	4	−3.02
2	1.000	0.750	1.000	1.000	0.667	1.000	0.889	1	−1.02
3	0.488	0.000	0.257	0.494	0.333	0.402	0.410	6	−7.74
4	0.700	1.000	0.400	0.625	1.000	0.455	0.693	5	−3.18
5	0.726	0.750	0.886	0.646	0.667	0.814	0.709	3	−2.99
6	0.000	0.000	0.000	0.333	0.333	0.333	0.333	9	−9.54
7	0.774	1.000	0.400	0.688	1.000	0.455	0.714	2	−2.92
8	0.014	0.250	0.314	0.336	0.400	0.422	0.386	7	−8.27
9	0.099	0.000	0.000	0.357	0.333	0.333	0.341	8	−9.34

**Table 8:** Response table for means of GRG.

Larger is better			
Level	Cutting height (mm)	Feed force (*N*)	Cutting speed (m/s)
1	**0.6683**	**0.7045**	0.4752
2	0.5785	0.6613	0.4814
3	0.4805	0.3615	**0.7707**
Delta	0.1878	0.3430	0.2956
Rank	3	1	2

### ANOVA for GRG

An analysis of variance (ANOVA) was performed based on the GRG values to determine the contribution of working parameters (Table 9). The ANOVA results revealed that the percentage contribution of the GRG values for cutting height, feed force, and cutting speed were 15.43%, 61.04%, and 19.58%, respectively. This indicated the relative importance of each factor in influencing the overall performance. According to both the rank analysis and the ANOVA results, the factors were ordered in terms of their significance as follows: B > C > A. Therefore, the feed force had the greatest influence on the GRG value, followed by the cutting speed and the cutting height. Taking into consideration the rank analysis and the analysis of variance, the optimal combination of parameters for maximizing the GRG value was determined to be a cutting height of 15 mm, a feed force of 10 N, and a cutting speed of 5 m/s. The regression model displayed a coefficient of determination (R^2^) of 0.960, signifying that 96.0% of the variation in GRG could be accounted for by the model. The adjusted R^2^value of 0.8422 indicated that approximately 84.2% of the variability in GRG could be explained by the independent variables incorporated in the model. These high R^2^ values indicate that the regression model has a strong explanatory power and is capable of predicting the relationship between the independent variables and the dependent variable (GRG) with a high level of accuracy.

**Table 9:** Analysis of variance for grey relational grade (GRG).

Source	df	Adj SS	Adj MS	Contribution
Cutting height (mm)	2	0.05294	0.026469	15.43%
Feed Force (N)	2	0.10546	0.052732	61.04%
Cutting speed (m/s)	2	0.06715	0.033576	19.58%
Error	2	0.01354	0.006769	3.95%
Total	8	0.34303		100.00%
Model Summary	S	R-sq	R-sq(adj)	
	0.082273	96.05%	84.21%	

### Confirmation test

The initial and ideal parameters acquired by using the Taguchi technique with GRA are shown in Table 10. Remarkable improvements were observed in the cutting force, SFL, and cutting time, with reductions from 57.57 to 46.7 N, level 3 to level 1, and 1.63 to 1.62 s, respectively. These results indicate significant reductions in cutting force and SFL, leading to enhanced cutting performance. In this study, the grey relational grade improved significantly from 0.569 to 0.891 as a result of the integration of the Taguchi approach with GRA. In comparison to the initial combination, A2B2C2, Table 10shows that the GRG value increased by 56.6% at the ideal combination, A1B1C3. Researchers have also utilized sophisticated methodologies such as Bayesian Optimization with Long Short-Term Memory (Bo-LSTM) and the Parameter-Weight-Adaptive Convolutional Neural Network (PWA-CNN) for the real-time prediction and optimization of cross-sectional characteristics and initial wrinkle formation in the tube bending process^54–57^. The combined use of these methods effectively contributed to the improved efficiency and performance of the cutting mechanism, offering practical implications for the optimization of cutting processes in the development of selective cauliflower harvester.

**Table 10:** Results of cutting performance using initial and optimal settings.

Parameters	Initial operating setting	Optimal operating setting	
		Prediction	Experiment
Level	A2B2C2	A1B1C3	A1B1C3
Cutting force	57.57		46.70
SFL	3		1
Cutting time	1.63		1.62
GRG	0.569	0.992	0.891
Improvement in GRG		74.3%	56.6%

## Conclusion

The newly developed cutting mechanism, based on the chainsaw cutting principle, displayed improved performance in terms of reduced cutting force and time requirements compared to impact and slicing methods for cauliflower harvesting. To enhance the harvesting performance of the selective cauliflower harvester, attention is directed towards optimizing the working parameters—namely cutting height, feed force, and cutting speed—of its chain saw cutting mechanism and examining their effects on response variables, including cutting force, splitting failure levels, and cutting time. Employing the Taguchi L9 orthogonal array in conjunction with GRA methodology facilitated a comprehensive exploration of the effects and contributions of these working parameters on multiple response parameters. The settings for the operation were optimized using the Taguchi technique with GRA to simultaneously minimize the cutting force requirement, splitting failure levels, and cutting time. The best parameter combination, designated as A1B1C3, was found by analysis of the GRG response table. This combination corresponds to a cutting height of 15 mm, a feed force of 10 N, and a cutting speed of 5 m/s. The ANOVA and GRG analyses revealed that the feed force parameter makes a significant contribution, accounting for the largest share (61.04%) in reducing cutting force, splitting failure levels (SFL), and cutting time. This is followed by cutting speed (19.58%) and cutting height (15.43%). Verification testing confirms that the ideal parameter combination yields a substantial improvement, evidenced by a 0.322 rise in GRG values, representing a 56.6% enhancement relative to the initial setting, A2B2C2.

These findings underscore the utility of this approach in enhancing the performance of the cutting mechanism. As a result, the optimal working parameter settings are included in the selective cauliflower harvester or development of selective harvesting modules which are operated by a tractor, promoting efficient and successful cauliflower harvesting processes. Thus, this improved cutting mechanism can inspire the farm machinery and tractor industries to advance mechanized harvesting of horticultural crops, especially cole crops, where mechanization levels have remained extremely low.

## Data Availability

The datasets generated during and/or analyzed during the current study are available from the corresponding author on reasonable request.
